# Dentition, and Its Connection with the Bowel-Affections of Children

**Published:** 1863-10

**Authors:** N. S. Davis

**Affiliations:** Chicago, Ill.


ARTICLE XXVIII.

### DENTITION, AND ITS CONNECTION WITH THE BOWEL-AFFECTIONS OF CHILDREN.

By N. S. DAVIS, M.D., Chicago, Ill.

Read to the Chicago Medical Society Sept. 11, 1868.

My object in calling the attention of the Society to a subject so familiar to every member, is to start an influence, if possible, against one of the most prevalent and mischievous popular errors of the day. The error to which I allude consists in attributing to the influence of the first dentition most of the attacks of cholera infantum, diarrhœa, dysentery, etc., that occur in children during every summer. It is well known that the diseases named occur as regular endemics in all the populous towns and cities in this country, during the summer months; that they effect all classes and ages, more or less; but that the two first are much more frequent and severe in children under two years of age than at any other period of life. In this latitude, the attacks of cholera morbus and diarrhœa, in children, usually begin early in July, and continue to increase in frequency throughout that month, although the highest ratio of mortality is in August. Notwithstanding the acknowledged endemic character of these diseases, and the general limitation of their prevalence to the hot months of the year, yet you will find, at least, four cases out of five attributed to "teething." This general assignment of *teething* as the cause of gastric and intestinal derangements, would be of little consequence, were it not for the almost universal neglect of proper treatment in the early stage, which it produces. No incident in practice is more common, during the summer and autumn, than to meet with young children, extremely pale, emaciated almost to the condition of skeletons, and yet having from five to fifteen intestinal evacuations per day, with occasional vomiting. You ask the mother how long the child has been sick? and the answer will generally be: "Oh, its bowels have been out of order all summer; but it has not been, to say, *very sick* until the last week."

You inquire what she has done for the child, and the ready answer will be: "I've done nothing at all, doctor. The child is *teething*, and I thought it would not signify, until the last few days, it has got so weak, I feared it would die." If you push the inquiry further, and ask if she has really let her child go so long with diarrhœa, without giving any medicine or asking any medical advice, she will acknowledge that she has given it castor oil or rhubarb, and sometimes even pink and senna. If you ask, why she thought the growth of the teeth caused the sickness, she will either immediately force the little sufferer's emaciated jaws open and triumphantly point you to the teeth that have appeared, and to the gums which are still enlarged by the advancing teeth; or she will say, that she consulted Dr. A. or Dr. B., and he told her "*it was the teeth*, and that it would not do to stop the diarrhœa *too soon*."

We think no observing physician, in active practice, can have failed to notice the very large number of children, especially among the poorer classes, in whom attacks of summer diarrhœa are entirely neglected until the stage of extreme emaciation and exhaustion has arrived, solely under the idea that such diarrhœa is caused by the growth of the teeth. From direct personal observation, I am satisfied that more than one hundred children, under two years of age, are regularly sacrificed in this city by such neglect during every returning summer and autumn. I mean that more than one hundred children die every season, in this city, from the neglect of the earlier stages of bowel-affections; the excuse for such neglect being that the children were "teething." If this is so, it becomes a very important question, how far the profession is responsible for the existence of the excuse and its consequences? Whether we look into the chapter on the diseases of dentition, contained in most of our works on practical medicine, or listen to the answers given to mothers daily by practitioners, concerning the ailments of children, we shall easily perceive that the chief responsibility in this matter rests directly on our profession. Thirty years since, when I was a student of medicine, it was thought to be as necessary to have a gum-lancet in the pocket as medicine in the medicine-case.

But is it true that the simple natural growth of the teeth frequently produces disease or disturbance of function either in the alimentary canal or elsewhere? To answer this question satisfactorily, we must enquire, first, whether there is anything in the structure of the gum or investing membrane of the tooth, or anything pertaining to their physiology, that would give them extensive sympathetic relations with other parts of the system? second, whether direct clinical observation has shown a frequent coincidence between actual irritation of the gums and bowel-affections? and, third, whether such bowel-affections are generally relieved or cured by any kind of treatment applied to the gums?

In relation to the first question, every anatomist knows that the structure of the gums is composed chiefly of elastic fibrous tissue, with only a moderate supply of capillary bloodvessels, and a still more sparing supply of sensitive nerves. In other words, they belong to the class of dense fibrous structures of low sensibility, and, physiologically, designed to bear pressure and, in a measure, protect the bodies of the teeth in mastication. The only nerves which could give them sympathetic relations with other organs are the branches of the fifth pair, by which they are supplied with sensibility, and the recognized diseases of which certainly do not often cause disturbance of the bowels. Facial and maxillary neuralgias are abundantly common at all periods of human life; and well-known irritation of the investing membrane of the teeth is among the common annoyances of all classes, but I have never yet found a case of the kind productive of sympathetic irritation of the bowels or diarrhœa. Hence, I am compelled to believe that there is nothing in either the anatomy or the physiology of the gums or dental periosteum which could explain the frequent occurrence of diarrhœa in infancy.

Does direct clinical observation show the frequent coincidence of irritation of the gums and diarrhœa in young children? To answer this question, we must first determine what constitutes evidence of irritation in the gum during teething. It will not be denied by anyone that the growth of the tooth and the gradual enlargement of the gum are strictly physiological processes, and not of themselves evidences of irritation; yet nothing is more common than to find simple enlargement alone presented, not only as evidence of irritation, but as a sufficient reason for scarification; and, of course, as a full and satisfactory cause of whatever derangement or disease may be coexisting at the time; and, inasmuch as there is scarcely any period of time, between the ages of six months and two years, that some of the gums in a child's mouth are not enlarged from advancing teeth, it is easy for either mother or physician to find this condition always present to account for each attack or aggravation of disease that may come to pass. In addition to simple enlargement, the next most frequent evidence of irritation is the disposition of the child to press or bite whatever is put into its mouth. Every practitioner is familiar with the common expression of mothers and nurses, in reference to the causes of illness in their children, "we are sure it is their teeth," say they, "for the gums are not only swelled, but the child is constantly putting its fingers in its mouth, and it bites everything it gets between its teeth." It requires but little observation to satisfy any intelligent physician that this disposition to put things into the mouth and press them with the gums is an instinctive habit of all children in the most perfect health; and, instead of indicating the existence of irritation, affords direct proof that no such condition exists. If any member of this Society has had irritation of his gums or of the periosteum of his teeth, the last thing he was willing to do was to bite, even so softly, on the affected parts. If we ask for the ordinary evidences of local irritation, such as heat, redness and tenderness, we shall rarely find any one of them present in the gums in the bowel-affections of children, except in such cases as are accompanied by fever sufficient to render the whole mouth and all other parts hotter than natural, and such as are accompanied by general inflammation of the mucous membrane of the mouth and fauces. For the purpose of gaining reliable information on this subject, I took the trouble to keep a record of fifty cases of diarrhœa in children under two years of age, during the months of July and August.

The record embraced cases in which the diarrhœa had continued from three days to six weeks, and was of almost every grade of severity. In twenty cases, the gums were *paler* than natural, corresponding with the general palor or anæmic condition of the patients, and entirely free from unnatural heat or tenderness. In twenty-four cases, the color of the gums was natural, without any increased heat or tenderness. In six cases, the gums, together with the whole mucous membrane of the mouth, were more red, more hot, and more dry than natural; and, in three of them, there was apthous ulcerations on the inner surface of the lips and cheeks. In all the six cases, there was general febrile action; and no difference could be perceived between the redness and temperature of the gums enlarged by advancing teeth and those where no such enlargement had taken place. The results afforded by this record are in strict accordance with my observations throughout a professional experience of twenty-seven years; and, if we set aside the simple fact of enlargement of the gums and the disposition of the child to bite on them, I am satisfied that the closest scrutiny will fail to find any other evidences of irritation in them, in ninety out of every one hundred cases of ordinary bowel-affections or sumner complaints of children. But there is another idea in relation to the effect of the growth of the teeth during dentition, which is contrary to the well-known laws of physiological growth. I allude to the idea, that, as the tooth advances, the investing membrane and gum becomes stretched or tense over it, and that such tension is the cause of irritation. It should be remembered that the parts investing a growing tooth are themselves also constantly undergoing change by absorption and nutrition, by which they maintain their relation to the enclosed tooth in every stage of its advancement. During the first fifteen years of my practice, I scarified the gums in several hundred cases; but in no one of them did I ever see the edges of the incision, made over the top of the tooth, recede from each other, as though the tissue was stretched or tense. Indeed, there is no more propriety in supposing the parts investing a tooth are put upon the stretch by its growth, than that the pericranium and scalp become tense by the growth of the skull.

Does the result of treatment directed to the gums, during the progress of dentition, afford evidence that they are the seat of irritation? In answer to this inquiry, I will simply state the results of my own observations:—When I commenced the practice of medicine, little more than twenty-seven years since, a gum-lancet in the pocket of the practitioner was deemed as necessary as medicine in his saddle-bags; and on almost all occasions of any kind of sickness in young children, if any of the gums were found enlarged simply, it was deemed proper to incise the gums. Consequently, during the first fifteen years of my practice I scarified the gums of children in a large number and variety of cases. During the last ten or twelve years, I have applied the knife to the gums in comparatively very few cases, but have met with many in which the operation had been performed by others. With all these opportunities for observation, I must say, that I have never seen a case of diarrhœa, dysentery, or cholera morbus relieved, in any degree, by any amount of incising or scarifying of the gums alone. Many cases have occurred in which the gums have been cut, and ordinary internal treatment directed at the same time, with speedy relief to the patient; but abundant trials have shown that the same internal treatment, without touching the gums, has been followed by the same results; while not a single instance of the reverse has come under my observation.

It is only a few days since that my attention was called to a child ten months old, pale, anæmic, emaciated almost to a skeleton from diarrhœa, which had continued since the middle of July. Its gums had been scarified four times by a physician in the neighborhood; and he was only deterred from making a fifth trial of the same remedy by the refusal of the mother, on the ground that the previous trials of it had done no good. We thus see that the extremely prevalent idea, that the first dentition is *the cause* of bowel-affections in young children, is not supported either by the anatomy and physiology of the gums and teeth, or by clinical observations at the bedside, or by the results of treatment. Indeed, when we remember that these affections are limited in their prevalence almost exclusively to the hot months of the year, and to certain geographical limits, we wonder how the idea, that the growth of the teeth acts any prominent part as a cause, could have gained even a respectable degree of credit; for, surely children cut their teeth as often in winter as in summer, in the country as in the city, and as often in one part of the globe as an other.

The remainder of the article relating to the real causes and pathology of the bowel-affections of children, and a glance at the general principles of treatment will be given in the next number of the *Examiner.*

